# From Sarcopenia to Depressive Symptoms in Elderly: A Path Analysis

**DOI:** 10.3390/ijerph20020972

**Published:** 2023-01-05

**Authors:** Cedomir Ustevic, Nina Rajovic, Dejana Stanisavljevic, Danijela Tiosavljevic, Andrija Pavlovic, Radica Tasic, Tatjana Rajovic, Jovana Grupkovic, Filip Pilipovic, Vedrana Pejin, Petar Milcanovic, Sanja Mazic, Natasa Milic

**Affiliations:** 1 Special Hospital for Medical Rehabilitation “Termal”, 22408 Vrdnik, Serbia; 2 Laboratory for Sports Institute of Medical Physiology, Faculty of Medicine, University of Belgrade, 11000 Belgrade, Serbia; 3 Institute for Medical Statistics and Informatics, Faculty of Medicine, University of Belgrade, 11000 Belgrade, Serbia; 4 Department of Humanities, Faculty of Medicine, University of Belgrade, 11000 Belgrade, Serbia; 5 Clinic for Psychiatry, University Clinical Centre of Serbia, 11000 Belgrade, Serbia; 6 Medical School, College of Vocational Studies, 11080 Belgrade, Serbia; 7 Department for Orthopedic and Trauma Surgery, Clinical Centre of Serbia, 11000 Belgrade, Serbia; 8 Institute for Orthopedic Surgery “Banjica”, 11040 Belgrade, Serbia; 9 Clinical Centre of Serbia, 11000 Belgrade, Serbia; 10 Department of Internal Medicine, Division of Nephrology and Hypertension, Mayo Clinic, Rochester, MN 55902, USA

**Keywords:** sarcopenia, malnutrition, quality of life, depression, elderly, path analysis

## Abstract

Background: Sarcopenia is an age-related progressive, generalized skeletal muscle disorder involving the accelerated loss of muscle function and muscle mass. The aim of this study was to assess the complex relationship between sarcopenia, malnutrition, cognitive impairment, physical activity, and depression in the elderly, with the potential role of quality of life as a mediator in these associations. Methods: A cross-sectional study was conducted on a sample (*n* = 298) of elderly patients admitted to Special Hospital for Rehabilitation “Termal”, Vrdnik, Serbia. Sarcopenia, the risk for malnutrition, cognitive impairment, physical activity, quality of life, and depressive symptoms were measured by standardized instruments. Additional data included sociodemographic characteristics. Simultaneous assessment of the direct and indirect relationships of all determinants was performed by path analysis. Results: A total of 40% (*n* = 120) of the elderly were diagnosed with sarcopenia, and 42.6% had depression symptoms. The risk of malnutrition was present in 23.5%, cognitive impairment in 5.4%, and a low level of physical activity was reported in 26.2% of elderly participants. The mean reported quality of life measured by Sarcopenia and Quality of Life Questionnaire was 60 (on the scale ranging from 0 to 100; where a higher score reflects a higher quality of life). The best-fitted model (χ^2^/DF = 1.885, NFI = 0.987, CFI = 0.993, GFI = 0.997, RMSEA = 0.055) highlighted the mediating effect of quality of life between sarcopenia, malnutrition, cognitive impairment, lower level of physical activities and depression. According to the model, quality of life was a direct negative predictor of depressive symptoms in the elderly, while malnutrition positively affected depression. Conclusions: The presented path model may assist rehabilitation centers in developing strategies to screen for sarcopenia and risk of malnutrition, and promote physical activity in elderly, aiming to prevent their negative effects on mental health. For the elderly currently affected by sarcopenia, we consider regenerative medicine and stem cell therapy, which, in view of their etiology, could be a potential therapeutic strategy for sarcopenia.

## 1. Introduction

Sarcopenia, first described as an age-related decline in lean body mass affecting mobility, functional independence, and nutritional status [1], has since evolved and has been marked by two important milestones. The introduction of muscle function into the concept was the first turning point, where focus on muscle strength and reduced physical performance occurred [2]. Recognition of sarcopenia as an independent condition represented the second milestone [3]. Muscle function has been shown as a more powerful predictor of clinically significant outcomes in elderly than muscle mass alone, and the muscle strength is considered the most reliable parameter to measure muscle function [4,5,6]. In addition, morphological insights into skeletal muscle composition suggested that fat accumulation within skeletal muscle is associated with lower muscle strength and increased risk of incident limitations in mobility [7,8], as well as poor function and increased risk of incident falls in elderly [9,10]. The most recent definition introduced by the European Working Group on Sarcopenia in Older People (EWGSOP2) in 2019 defines sarcopenia as a progressive, generalized skeletal muscle disorder, involving the accelerated loss of muscle function and muscle mass or quality [6].

Although a significant number of studies investigating the prevalence of sarcopenia have been conducted, the results are inconsistent ranging from 5.2% to 41% and increases with age [11,12]. The vast difference in found prevalence can be attributed to an undetermined global definition of the illness, and unclear diagnostic criteria, problems that the updated definition (EWGSOP2) aimed to address [6]. Up until recently, sarcopenia was mainly observed as a part of clinically well-recognized frailty syndrome, which is defined as a reduced physiologic reserve vulnerable to external stressors [13]. Whether we perceive it independently or as a part of frailty syndrome it is well documented that it has rapid progression, and affects the patient’s quality of life immensely; it increases chances of a fall by 2-fold, as well as fracture risk, hospitalization, and mortality, and it is associated with functional disabilities, cognitive decline, osteoporosis, dyslipidemia, immunosuppression, an increased cardiovascular risk, and metabolic syndrome [14,15,16,17]. As such, if left undiagnosed and untreated it can represent a significant personal, social, and financial burden [18].

The association of sarcopenia and various physical adverse effects had been proved by a growing body of literature, but its causal relationship with mental health in elderly remains poorly understood [19]. Depression, a leading mental health disorder worldwide, is shown to be linked to an increased likelihood of numerous health problems among the elderly, with its prevalence going up to 38% [20]. Association between depression and sarcopenia appeared inconsistent according to the literature; while Hsu et al. [21] proposed that sarcopenia was linked with depression, Byeon [22] reported no high prevalence of depression in patients with sarcopenia. In addition, a systematic review and meta-analysis, found that sarcopenic patients were more prone to developing depressive symptoms, but concluded that this relationship needs further validation [19]. Furthermore, pathophysiology of sarcopenia includes physical inactivity and malnutrition [23], conditions common in the elderly and often jointly associated with depression [24].

In mainstream practice, sarcopenia has been largely undertreated and overlooked, due to the complexity of the condition and the diagnostic tools needed to identify it [25]. Although the growing interest of medical community resulted in the development of several clinical practice guidelines for sarcopenia, the applicability of guidelines for clinical practice was granted lowest score in a study examining their quality [26]. Therefore, the aim of this study was to assess the complex relationship between sarcopenia, malnutrition, cognitive impairment, physical activity, and depression in the elderly, with the potential role of quality of life as a mediator in these associations, using path analysis, on a sample of elderly patients admitted at Special Hospital for Rehabilitation in Serbia.

## 2. Materials and Methods

### 2.1. Study Design

This was a cross-sectional study conducted on a sample of elderly patients admitted at the Special Hospital for Rehabilitation “Termal”, Vrdnik, Serbia, in the period from September 2019 to December 2019. Ethical approval was obtained from the Institutional Review Board (IRB) of the Special Hospital for Rehabilitation “Termal”, Vrdnik, Serbia (ethical code: 677/1, approved date: 19 March 2019). All participants were adequately informed of the purpose and benefits of this study, the way the study will be realized, the requests and risks the study poses to the participants, and the right of the participant to stop and refuse to continue participation in the study at any moment starting from the beginning to the end of the study, without giving reason(s) for withdrawing and oral informative consent was obtained and documented in the records at the beginning of the study [27]. The study was conducted in accordance with STROBE guidelines for cross-sectional studies [28].

Participants were included in the study if they were 65 years or older, native Serbian speakers, and were able to comprehend and complete questions and tasks related to the study. Participants were excluded if they were immobilized, had an amputated limb or had a limb deformity limiting their mobility, suffered from an acute disease, or from any neuropsychiatric disorder that could influence their collaboration.

Sociodemographic and medical history characteristics such as age, sex, smoking status, educational status, marital status, driving status, living place, data regarding household members, and comorbidities reported by the participant, were recorded by study coordinator. Presence of sarcopenia, risk for malnutrition, cognitive impairment, level of physical activity, quality of life and presence of depressive symptoms were assessed by a multidisciplinary team of physicians, consisting of physical rehabilitation specialists, psychiatrist, neurologist, and epidemiologist, by using following standardized instruments:

### 2.2. Strength, Assistance with Walking, Rising from a Chair, Climbing Stairs, and Falls Questionnaire (SARC-F) [29]

A SARC-F Questionnaire is a self-reported tool used for the initial screening of patients for possible sarcopenia. The questionnaire is comprised of 5 components: strength, assistance with walking, rising from a chair, climbing stairs, and experiencing falls. Each of the components was assigned numerical values from 0 to 2 to indicate the degree of severity. The total score range of the questionnaire is 10, with a SARC-F score of ≥4 predicting sarcopenia.

### 2.3. Short Physical Performance Battery (SPPB) [30]

The Short Physical Performance Battery (SPPB) is a predictive tool used to assess physical performance and possible disability in older patients. It comprises gait speed, chair stand, and balance tests. Each test is weighted with a score between 0 and 4 points, with a total score of 12. A score of 10 or greater indicated no sarcopenia, scores ranging from 3 to 9 indicated possible sarcopenia, and scores of 2 or lower indicated sarcopenia.

### 2.4. Sarcopenia and Quality of Life Questionnaire (SarQol) [31,32]

The SarQol is a self-administrated, 55-item questionnaire specific to sarcopenia, used to measure the quality of life of elderly subjects aged 65 years and older. Items consisted in the questionnaire measure seven domains of health-related quality of life: Physical and Mental Health (8 items), Locomotion (9 items), Body Composition (3 items), Functionality (14 items), Activities of daily living (15 items), Leisure activities (2 items), and Fears (4 items). Overall scores of the 55 items ranged from 0 to 100. A higher score reflected a higher quality of life.

### 2.5. The 36-Item Short Form Health Survey (SF-36) [33]

In order to measure the impact of health on an individual’s everyday life, a self-administered SF-36 questionnaire was used. Eight domains of functioning were assessed: Physical functioning, physical role functioning, bodily pain, general health perceptions, vitality, social role functioning, emotional role functioning, and mental health. Overall scores of the 36 items ranged from 0 to 100. A higher score was associated with a higher quality of life. Additionally, physical and mental health summary scales were calculated.

### 2.6. The EuroQOL Five-Dimension Questionnaire (EQ-5D) [34]

The EuroQOL Five-Dimension Questionnaire (EQ-5D) is a self-reported instrument used to assess the function of the following five domains: mobility, self-care, usual activities, pain/discomfort, and anxiety/depression of the respondents. Each of the domains comprises 3 levels indicating: no problems, some problems, and extreme problems. In addition, a Visual Analogue Scale (VAS) is used to record the self-rating health of the respondent ranging from 0 (worst imaginable health state) to 100 (best imaginable health state).

### 2.7. Geriatric Depression Scale (GDS) [35]

The Geriatric Depression Scale (GDS) is a 15-item self-report scale used to identify the presence of depression. Participants were asked to answer yes or no about how they felt over the past week. Of the 15 items, 10 items, when answered positively, indicated the presence of depression, and 5 items, when answered negatively, indicated depression (question numbers 1, 5, 7, 11, 13). A score ≥ 5 indicated depression.

### 2.8. Mini Nutritional Assessment (MNA) [36]

The Mini Nutritional Assessment (MNA) is a self-administrated nutrition screening tool used to identify geriatric patients aged 65 and above who are at risk of malnutrition. It consists of 18 items, comprising anthropometric measurements and questions regarding dietary intake, global assessment nutrition, and self-assessment nutrition. The score ranges from 0 to 30, with scores lower than 17 indicating malnutrition.

### 2.9. Mini-Mental State Examination (MMSE) [37]

In order to test cognitive impairment among the elderly, the Mini-Mental State Examination (MMSE) was assessed (PAR Order—10854035). The questionnaire includes tests of orientation, immediate recall, calculation/attention, delayed recall, naming, repetition, three-stage command, reading, writing, and constructional praxis. The total score is 30, with scores lower than 24 indicating cognitive impairment.

### 2.10. International Physical Activity Questionnaire (IPAQ) [38]

In order to measure physical activity among participants, International Physical Activity Questionnaire (IPAQ) was assessed. IPAQ is a self-reported questionnaire, proposing three levels (categories) of physical activity: high, moderate, and low physical activity. Additionally, continuous scores were measured in MET-min per week: MET level × minutes of activity × events per week.

### 2.11. Grip Strength

Additionally, grip strength was measured using the hand-held Jamar dynamometer, a validated and commonly used tool. With the elbow straight in a standing position, the patient squeezes the dynamometer using all of their strength. The test is repeated three times with each hand, and the maximal strength on trials for each hand gives an average score. If the score was <26 kg and <16 kg for men and women, respectively, the condition can be characterized as sarcopenia.

### 2.12. Sarcopenia

Sarcopenia was defined in patients who tested positive for sarcopenia based on: (1) SARC-F Questionnaire, (2) Short Physical Performance Battery, or (3) Jamar dynamometer.

### 2.13. Statistical Analysis

Numerical data were summarized as mean with a standard deviation of 95% Confidence Interval (CI). Categorical variables were presented by absolute numbers with percentages. Categorical variables were analyzed by the chi-square test. The Independent Sample T-Test and The Mann–Whitney U-test were applied for continuous variables based on a normal distribution analysis. Path analysis was used to test the hypothetical effects of sociodemographic characteristics, sarcopenia, cognitive impairment, physical activity, and quality of life of the participants on depressive symptoms and the mediation effects of quality of life on depressive symptoms. Univariate and multivariate logistic regression models were used to assess predictors of depression (as dependent variable). Path analysis was conducted as it allows the estimation of the direct and indirect effects of the multiple determinants through the simultaneous modeling of related regression relationships [39]. Multiple measures were used to assess the proposed model, which includes the χ^2^ test and the following fit indices: The Goodness of Fit Index (GFI), the Tucker- Normed Fit Index (NFI), the Comparative Fit Index (CFI), and the Root Mean Square Error of Approximation (RMSEA). The model consistency was evaluated by the χ^2^ test, which indicates, when nonsignificant, that the data are consistent. CMIN/DF test is proposed as a measure of fit instead of test statistics, indicating suitable fit when its value is less than two times its degree of freedom. The acceptable model fitting values for fit indices were defined as follows: GFI ≥ 0.95, NFI ≥ 0.95, CFI ≥ 0.95, RMSEA < 0.05. In the model, the arrows demonstrate the direction of the hypothesized association. Path estimates were presented in the form of standardized regression coefficients, which demonstrate the strength of the path between variables. To enable comparison between variables, these standardized path coefficients were estimated to be on a common scale ranging from −1 to 1. The direct coefficient in the model demonstrates the effect of independent characteristics on a dependent variable after controlling for other predictors in the path model, whereas the indirect coefficient presents the effect of independent characteristics on a dependent variable that is mediated by other variables on the path. The total effect between the two variables is a sum of the direct and indirect effects. The significance level was 0.05 in all analyses. Statistical analysis was performed by IBM SPSS Statistics 25 and Amos 21 (IBM SPSS Inc., Chicago, IL, USA, 2012).

## 3. Results

A total of 298 participants were enrolled in the study. Most of the participants were female (77.5%) with an average age of 70.8 ± 5.4 years. Almost half of the participants had a second level of education (49.8%) and were married (49.0%). Two-thirds of the respondents were living in a house (66.1%), mostly with two members in the household (39.2%). The majority of the participants had children (92.6%) and grandchildren (87.8%), with 61.1% taking care of their grandchildren on a regular basis. Thirty-nine participants were smokers (13.1%), and 31.7% were drivers. The sociodemographic characteristics of the study population are presented in Table 1.

A total of 40% (*n* = 120) of the elderly were diagnosed with sarcopenia, and 42.6% had depression symptomatology (Table 2). Both sarcopenia and depression symptomatology was present in one-fifth of the study population (19.8%). A low level of physical activity on the IPAQ was reported in 26.2% of participants, a moderate level in 38.3%, and a high level of physical activity reported in 35.6% of participants. Cognitive impairment was reported in 5.4% of participants based on the MMSE questionnaire. The risk of malnutrition was present in 23.5% of the study population, according to the MNA questionnaire. The mean reported quality of life measured by the Sarcopenia and Quality of Life Questionnaire was 60 (95% CI: 58.6−61.4) (on a scale ranging from 0 to 100, where a higher score reflects a higher quality of life). The mean reported quality of life according to the SF-36 questionnaire was 53.1 (95% CI: 50.9−55.4). According to the EuroQol Questionnaire, some problems with mobility were reported in 60.3% of participants, 74.1% with self-care, with usual activities 34.9%, 76.2% with pain/discomfort, and 18.8% with anxiety/depression (Table 2).

Multiple determinants assessed in a study according to the presence of depressive symptoms are presented in Table 2. Sarcopenia was more often present in depressive than in non-depressive patients (*p* = 0.020). Depressive patients reported a lower quality of life compared to patients with no depression in the overall score of the SarQol Questionnaire (*p* < 0.001). In addition, depressive patients had lower scores on physical and mental summary scales, as well as lower overall scores on the SF-36 Questionnaire (*p* < 0.001 for all). The risk of malnutrition or malnutrition was more often found in depressive patients (*p* < 0.001). Physical activity measured in weekly MET was significantly lower in depression than in participants with no depression (*p* = 0.030). In addition, patients with depression more often had cognitive impairment (*p* = 0.007).

In univariate logistic regression analysis, female sex (*p* = 0.030), older age (*p* = 0.007), presence of sarcopenia (*p* = 0.021), risk of malnutrition (*p* < 0.001), and low physical activity (*p* = 0.032) were significant risk factors for depression, while higher cognitive ability (assessed by MMSE) and quality of life (assessed by SarQol) were protective factors (*p* = 0.009 and *p* < 0.001, respectively). Malnutrition and quality of life were significant predictors of depression in multivariate logistic regression analysis (*p* = 0.001 and *p* < 0.001, respectively) (Table 3).

**Figure 1:**
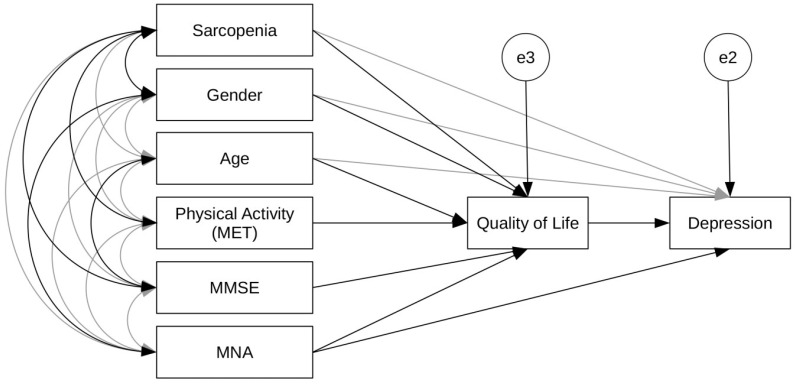
Path model of sociodemographic characteristics and quality of life of the participants on depressive symptoms (MMSE, Mini-Mental State Examination; MNA, Mini Nutritional Assessment).

The hypothesized relationships among the assessed characteristics of the elderly, tested by path analysis using a maximum likelihood estimate, are presented in Figure 1. The best fit of the constructed path model was achieved with χ^2^ = 3.770, df = 2, CMIN/DF = 1.885, *p* = 0.152, NFI = 0.987, CFI = 0.993, GFI = 0.997, and RMSEA = 0.055. B standardized coefficients were used in the model to estimate the variables predicting effects (Table 4). The presented path model accounted for 27% of the quality of life and 38% of depression. According to this model, quality of life and malnutrition were the main direct predictors of depression in the elderly (Figure 1). The elderly with a decreased level of self-rated quality of life and a higher risk for malnutrition had a higher level of depressive symptoms. In addition, the important mediating role of quality of life was also identified in the model. The female sex, older age, risk of malnutrition, cognitive impairment, lower level of physical activities measured in weekly MET, and sarcopenia had significant indirect effects on depressive symptoms via quality of life (Figure 1).

## 4. Discussion

This study aimed to address the complex relationship between sarcopenia, malnutrition, decreased physical activity, cognitive impairment, quality of life, and depressive symptomatology. Similar frequencies of sarcopenia and depression were found in our study (40% and 42.6%, respectively), with one-fifth of the study population having both (19.8%). In the constructed path model, increased depressive symptoms were significantly predicted by a lower level of self-rated quality of life and malnutrition in the elderly, while sarcopenia, cognitive impairment, and lower level of physical activities (measured in weekly MET) had important indirect effects on depressive symptomatology via the quality of life.

Sarcopenia is known to be associated with aging as well as numerous adverse health conditions such as cardiovascular diseases, diabetes, metabolic syndrome, and higher mortality [15,16]. The possibility of a link between mental health and sarcopenia was recently proposed [16]. Patients with depression are likely to be physically inactive and have an increased risk of malnutrition, conditions known to be associated with sarcopenia and poorer quality of life. Depression can coexist with sarcopenia, but whether sarcopenia directly affects symptoms of depression, apart from concomitant confounders, is still not resolved. Different studies have assessed the association between sarcopenia and quality of life. In a study by Patel et al. conducted in the United Kingdom, results showed that sarcopenic patients had a reduction in the quality of life, particularly in general health and physical functioning domains [40]. Beaudart et al. showed that sarcopenic individuals were found to have poorer quality of life, especially in domains of physical health and physical function [41]. This corresponds to our study results, where sarcopenia was in a negative correlation with the quality of life, which in turn directly influenced depressive symptomatology. Depression burdens people of all ages, particularly the elderly, who are less likely to seek treatment. One of the problems related to depression is the failure to recognize its symptoms and the feeling of helplessness usually present in the elderly. Depressive symptomatology tends to last longer in the elderly and frequently coexists with other medical disabilities and illnesses, especially with an increased risk of frailty [42]. Depression is potentially deadly because it is one of the leading causes of suicide, and people who commit suicide are especially those whose depression remains unrecognized and untreated [43].

Due to age-related modifications in body composition, the presence of sarcopenia is often side-by-side with malnutrition [44]. In our study, according to the MNA screening tool, the risk of malnutrition was present in 23.5% of the study participants. According to the literature, malnutrition is shown to be in relation to both sarcopenia and depression [45]. Results of the study conducted by Wang et al. [46] showed that scores of MNA were lower in depressive patients in comparison to non-depressed patients, which is in line with the results of our study. Han et al. [47] propose that the relationship between sarcopenia and depression may be mediated by malnutrition and that malnutrition can be an important confounding factor in this association. However, the causal relationship between malnutrition with depression remains still unclear. Literature data remains inconsistent; in the study conducted by Kvamme et al. [48], a strong independent association between malnutrition and depression was found, whereas other studies showed a modest association between nutritional deficit and depression [49] or no association at all [50]. In our study, malnutrition had both direct and indirect effects on depressive symptomatology.

Different studies examined a decline in physical activity in sarcopenic patients [51]. It has been reported that sarcopenia is associated with physical inactivity, poor endurance, frailty, slow gait speed, and decreased mobility. In addition, it is highly predictive of poor quality of life, incident disability, and all-cause mortality [52]. In our study, lower levels of physical activity were reported in 26.2% of the elderly. The latest EWGSOP2 definition even suggests that physical performance should be considered a measure of the severity of sarcopenia. According to EWGSOP2, physical performance is defined as the ability to accomplish physical tasks in order to be able to function independently in daily life [6]. Besides functional mobility, adequate cognitive performance and positive mood are necessary for the independent living of every individual. It is considered that the presence of cognitive impairment and depressive symptomatology in frailty syndrome is associated with a serious decline in functional health and carries out an increased risk of institutionalization and mortality [53]. In a study by Yuenyongchaiwat et al. [54], it was reported that the elderly with cognitive impairment were physically inactive and had higher depression levels compared to participants without cognitive impairment. Thus, depression is associated with poor cognitive performance, which often correlates to a lack of exercise or insufficient physical activity, which could be a contributing factor to sarcopenia. The results of this study confirm that depression could be a contributing factor to sarcopenia; however, sarcopenia could also contribute to increased depressive symptomatology, and in addition, lower physical activity might be linked both to sarcopenia and depression [54]. From this association, it appears that much of the prevalence of sarcopenia could be reduced or prevented by lifestyle modification, such as increased exercise or other physical activity. Positive effects of physical activity on muscle strength in the elderly were shown during the COVID-19 pandemic [55]. Our data clearly linked physical performance, cognitive impairment, and sarcopenia to the appraisal of depressive symptomatology through a mediating role of quality of life.

Future directions. Based on our findings, we propose that rehabilitation centers screen for sarcopenia at an early stage, aiming to initiate preventive interventions to avoid a further functional decline in the elderly. Given its multidimensional nature, a proper assessment tool should be used for sarcopenia screening, taking into account all of its domains. A case-finding approach has been recently proposed, which aims to screen for sarcopenia in healthcare settings, such as rehabilitation centers or nursing homes, where a higher prevalence of sarcopenia is expected. A frequently recommended case-finding instrument, such as the SARC-F Questionnaire, used in our study represents a fast and reliable tool that might be used to identify cases of sarcopenia in clinical practice [2,25]. The use of the SARC-F Questionnaire would help triage those patients who need more detailed diagnostics or specific interventions. Finding effective treatment strategies for sarcopenia should be a focus of future research. Currently, available treatment options involving the identification and modification of risk factors to prevent sarcopenia are suboptimal and of limited value to the elderly already affected by sarcopenia. Physical exercise, nutritional support, and pharmaceutical interventions are the cornerstone of current therapy, showing controversial results in sarcopenia alleviation. The development of effective therapies remains challenging due to the complex etiology of the disease. Regenerative medicine and stem cell therapy appear as attractive therapeutic intervention strategies for age-associated conditions and, in view of their etiology, could be a potential therapeutic strategy for sarcopenia [56,57].

Our study has several limitations. First, this study was conducted at a single center in Serbia, thus limiting the generalization of the results. The Special Hospital for Rehabilitation, “Termal”, maintains mostly patients from the territory of the autonomous province of Vojvodina, thus not representing other regions of Serbia. Future studies are needed to determine if similar results can be obtained in different clinical settings. Second, Dual-energy X-ray Absorptiometry (DXA), the gold-standard technique in the analysis of body composition, was not used in this study. Although DXA is the most widely used because of its low radiation exposure and modest scan cost, some pitfalls have been addressed in its measurement of muscle mass. It has been demonstrated that the quality of DXA is commonly influenced by errors in the acquisition, analysis, and interpretation, leading to inappropriate diagnosis and clinical decisions [58]. Third, sarcopenia prevalence may be overestimated in a hospital-based rehabilitation setting in contrast to the general population.

## 5. Conclusions

The present study has confirmed that sarcopenia may be linked to depression in the elderly. Low quality of life presented a significant risk factor for depressive symptoms in the elderly, while sarcopenia, cognitive impairment, and lower level of physical activities had important indirect effects on quality of life. Malnutrition had both direct and indirect effects on depression. The presented path model may assist rehabilitation centers in developing strategies to screen for sarcopenia and risk of malnutrition and promote physical activity in the elderly, aiming to prevent their negative effects on mental health. For the elderly currently affected by sarcopenia, we consider regenerative medicine and stem cell therapy, which, in view of their etiology, could be a potential therapeutic strategy for sarcopenia.

## Figures and Tables

**Table 1:** Sociodemographic characteristics of the study population.

Variable	*n* (%)
Age, mean ± sd	70.8 ± 5.4
Sex, female	231 (77.5)
BMI, mean ± sd	29.7 ± 4.8
Smoker, yes	39 (13.1)
Level of education	
Primary education or below	87 (29.7)
Secondary education	146 (49.8)
Tertiary education or above	60 (20.5)
Marital status	
Single	17 (5.7)
Married	145 (49.0)
Divorced	20 (6.8)
Widowed	109 (36.8)
Domestic partnership	5 (1.7)
Living place	
Apartment	100 (33.9)
House	195 (66.1)
Number of household members	
One	88 (30.2)
Two	114 (39.2)
Three or more	89 (30.6)
Having children, yes	277 (92.6)
Having grandchildren, yes	260 (87.8)
Taking care of grandchildren, yes	179 (61.1)
Driving status, yes	93 (31.7)

**Table 2:** Sarcopenia, quality of life, malnutrition, physical activity, and cognitive decline, total and according to depressive symptoms in the elderly.

Variable	Total (*n* = 298)	Depression		*p*-Value
		No (*n* = 167)	Yes (*n* = 124)	
**Sarcopenia**	***n* (%)**	***n* (%)**	***n* (%)**	
No	178 (59.7)	110 (65.9)	65 (52.4)	0.020
Yes	120 (40.3)	57 (34.1)	59 (47.6)	
**SarQol Questionnaire**	**mean (95% CI)**	**mean (95% CI)**	**mean (95% CI)**	
D1 (Physical and mental health)	62.7 (60.9−64.5)	69.1 (66.9−71.2)	53.9 (51.6−56.3)	<0.001
D2 (Locomotion)	55.7 (54.3−57.1)	58.8 (56.9−60.7)	51.2 (49.5−52.9)	<0.001
D3 (Body Composition)	62.4 (60.6−64.2)	67.4 (65.3−69.4)	55.5 (52.7−58.4)	<0.001
D4 (Functionality)	64.8 (63.1−66.6)	71.2 (69.0−73.3)	55.8 (53.6−58.0)	<0.001
D5 (Daily Activities)	56.2 (54.3−58.1)	62.7 (60.3−65.2)	46.9 (44.5−49.2)	<0.001
D6 (Leisure Activities)	32.1 (29.7−34.5)	35.6 (32.3−39.0)	27.5 (24.0−31.0)	0.001
D7 (Fears)	85.9 (83.9−87.8)	88.6 (86.1−91.1)	81.7 (78.8−84.7)	0.001
Overall Score	60.0 (58.6−61.4)	65.5 (63.9−67.2)	52.1 (50.5−53.8)	<0.001
**SF−36**	**mean (95% CI)**	**mean (95% CI)**	**mean (95% CI)**	
Physical functioning	61.6 (58.8−64.5)	70.0 (66.6−73.4)	49.9 (45.5−54.2)	<0.001
Physical role functioning	24.5 (20.0−29.0)	31.2 (24.6−37.8)	15.4 (9.6−21.3)	<0.001
Bodily pain	51.9 (49.2−54.7)	59.8 (56.0−63.6)	41.5 (38.1−44.8)	<0.001
General health perceptions	57.5 (55.4−59.6)	64.7 (62.4−67.1)	47.7 (44.6−50.7)	<0.001
Vitality	56.3 (54.0−58.5)	64.9 (62.2−67.7)	44.6 (42.2−47.1)	<0.001
Social role functioning	69.3 (66.6−72.0)	78.3 (74.9−81.6)	57.1 (53.4−60.9)	<0.001
Emotional role functioning	38.1 (32.9−43.3)	46.0 (38.8−53.1)	26.4 (19.1−33.7)	<0.001
Mental health	65.9 (63.8−68.0)	74.5 (72.2−76.8)	54.2 (51.3−57.1)	<0.001
Physical Health Summary Scale	50.4 (48.2−52.7)	58.2 (55.3−61.1)	39.8 (37.2−42.5)	<0.001
Mental Health Summary Scale	57.4 (55.2−59.6)	65.6 (63.0−68.2)	46.0 (43.2−48.7)	<0.001
Overall Score	53.1 (50.9−55.4)	61.2 (58.4−64.0)	42.1 (39.5−44.7)	<0.001
**EQ-5D Questionnaire**	***n* (%)**	***n* (%)**	***n* (%)**	
Mobility (reporting some problems)	179 (60.3)	100 (59.9)	74 (60.2)	0.961
Self-care (reporting some problems)	220 (74.1)	120 (73.3)	94 (75.8)	0.500
Usual activities (reporting some problems)	104 (34.9)	54 (32.3)	48 (38.7)	0.260
Pain/Discomfort (reporting some problems)	126 (76.3)	124 (74.7)	98 (79.7)	0.322
Anxiety/Depression (reporting some problems)	56 (18.8)	24 (14.5)	31 (25.0)	0.023
Visual Analogue Scale (VAS)	55 (52.3−57.7)	55.8 (52.1−59.5)	55.2 (51.4−59.1)	0.468
**MNA Questionnaire**	***n* (%)**	***n* (%)**	***n* (%)**	
Good nutrition	228 (76.5)	149 (89.2)	74 (59.7)	<0.001
Risk of malnutrition/Malnutrition	70 (23.5)	18 (10.8)	50 (40.3)	
**Physical activity (MET)**	**mean (95% CI)**	**mean (95% CI)**	**mean (95% CI)**	
	2905 (2554−3257)	3262 (2758−3766)	2461 (1965−2957)	0.030
**MMSE Questionnaire**	**mean (95% CI)**	**mean (95% CI)**	**mean (95% CI)**	
	28.84 (28.6−29.1)	29.2 (28.8−29.5)	28.4 (28.0−28.9)	0.007

SarQol, Sarcopenia and Quality of Life Questionnaire; SF-36, The 36-item Short Form Health Survey; EQ-5D, The EuroQOL Five-Dimension Questionnaire; GDS, Geriatric Depression Scale; MMSE, Mini-Mental State Examination; MNA, Mini Nutritional Assessment; SARC-F, Strength, assistance with walking, rising from a chair, climbing stairs, and falls Questionnaire; SPBB, Short Physical Performance Battery.

**Table 3:** Univariate and multivariate logistic regression analysis with depression as a dependent variable.

Variable	Univariate			Multivariate		
	*p*	OR	95%CI for OR	*p*	OR	95%CI for OR
Sex	0.030	1.918	1.065−3.454			
Age	0.007	1.066	1.017−1.117			
Sarcopenia	0.021	1.752	1.088−2.819			
MNA	<0.001	5.593	3.049−10.259	0.001	3.692	1.700−8.016
MMSE	0.009	0.869	0.783−0.965			
Physical activity, MET	0.032	1.001	1.000−1.001			
QoL	<0.001	0.872	0.843−0.902	<0.001	0.880	0.844−0.917

MMSE, Mini-Mental State Examination; MNA, Mini Nutritional Assessment.

**Table 4:** Direct, indirect, and total effects of multiple determinants on depressive symptoms in the elderly.

	B	SE	*p*
**Direct**			
Sarcopenia→QoL	−4.673	1.269	<0.001
MMSE→QoL	0.704	0.270	0.009
MET→QoL	0.001	0.000	0.013
MNA→QoL	−7.125	1.483	<0.001
Age→QoL	−0.336	0.115	0.003
Sex→QoL	−6.072	1.510	<0.001
Sarcopenia→GDS	0.158	0.351	0.653
MNA→GDS	1.620	0.419	<0.001
Age→GDS	0.010	0.032	0.753
Sex→GDS	−0.394	0.421	0.350
QoL→GDS	−0.158	0.015	<0.001
**Indirect**			
Sarcopenia→QoL→GDS	0.740	0.241	0.010
MMSE→QoL→GDS	−0.111	0.043	0.010
MET→QoL→GDS	0.001	0.000	0.024
MNA→QoL→GDS	1.129	0.235	0.010
Age→QoL→GDS	0.053	0.019	0.010
Sex→QoL→GDS	0.962	0.250	0.010
**Total**			
Sarcopenia→GDS	0.898	0.426	0.029
MMSE→GDS	−0.111	0.043	0.010
MET→GDS	0.001	0.000	0.024
MNA→GDS	2.749	0.541	0.010
Age→GDS	0.063	0.034	0.068
Sex →GDS	0.568	0.502	0.249
QoL→GDS	−0.158	0.016	0.010

Qol, Quality of Life; GDS, Geriatric Depression Scale; MMSE, Mini-Mental State Examination; MNA, Mini Nutritional Assessment; B, regression coefficient; SE, standard error.

## Data Availability

The data that support the findings of this study are available on request from the corresponding author.
